# 1-(10*H*-phenothia­zin-10-yl)ethanone

**DOI:** 10.1107/S1600536812045904

**Published:** 2012-11-17

**Authors:** Eri Tokunaga, Tsunehisa Okuno

**Affiliations:** a Department of Material Science and Chemistry, Wakayama University, Sakaedani, Wakayama 640-8510, Japan

## Abstract

In the title compound, C_14_H_11_NOS, the phenothia­zine unit has a butterfly conformation and the central six-membered ring has a boat form. The fold angle between the benzene rings is 46.39 (7)°, which is larger than found in similar compounds, probably as a result of steric repulsion between the phenothia­zine fragment and the acetyl group.

## Related literature

For the structures of related *N*-alkyl­phenothia­zine derivatives, see: Chu & Van der Helm (1974 ▶, 1975 ▶) and of related *N*-acetyl­phenothia­zine derivatives, see: Meester & Chu (1986 ▶); Wang *et al.* (2009 ▶); Siddegowda *et al.* (2011 ▶).
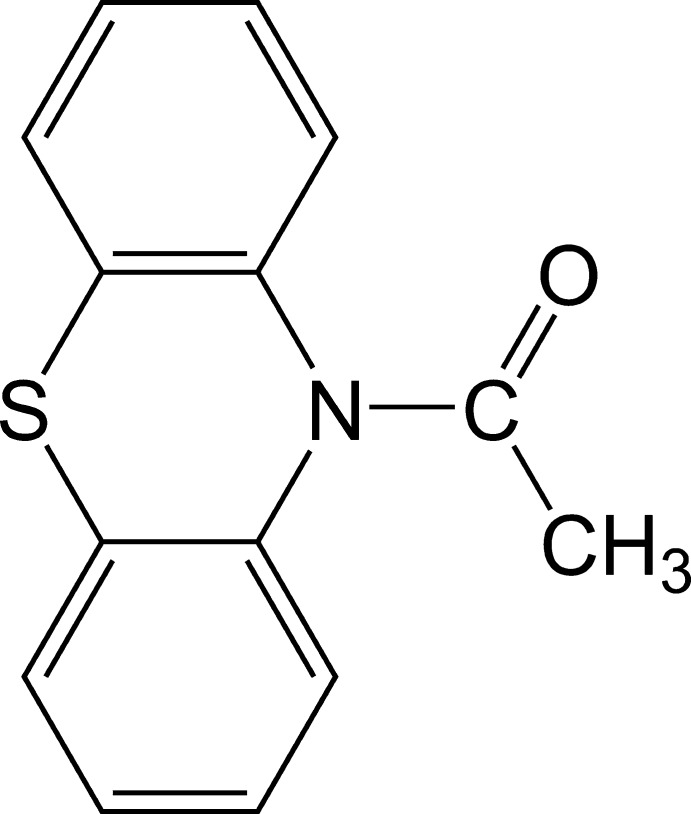


## Experimental

### 

#### Crystal data

C_14_H_11_NOS
*M* *_r_* = 241.31
Monoclinic, 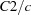

*a* = 21.435 (6) Å
*b* = 8.897 (3) Å
*c* = 12.738 (4) Å
β = 111.753 (3)°
*V* = 2256.2 (11) Å^3^
*Z* = 8
Mo *K*α radiation
μ = 0.27 mm^−1^
*T* = 93 K
0.17 × 0.10 × 0.10 mm

#### Data collection

Rigaku Saturn724+ diffractometer
Absorption correction: numerical (*NUMABS*; Rigaku, 1999 ▶) *T*_min_ = 0.969, *T*_max_ = 0.974
9030 measured reflections
2584 independent reflections
2337 reflections with *F* ^2^ > 2σ(*F* ^2^)
*R*_int_ = 0.025

#### Refinement

*R*[*F* ^2^ > 2σ(*F* ^2^)] = 0.031
*wR*(*F* ^2^) = 0.081
*S* = 1.03
2584 reflections
154 parameters
H-atom parameters constrained
Δρ_max_ = 0.31 e Å^−3^
Δρ_min_ = −0.24 e Å^−3^

### 

Data collection: *CrystalClear* (Rigaku, 2008 ▶); cell refinement: *CrystalClear*; data reduction: *CrystalClear*; program(s) used to solve structure: *SHELXS97* (Sheldrick, 2008 ▶); program(s) used to refine structure: *SHELXL97* (Sheldrick, 2008 ▶); molecular graphics: *ORTEP-3* (Farrugia, 2012 ▶); software used to prepare material for publication: *CrystalStructure* (Rigaku, 2010 ▶).

## Supplementary Material

Crystal structure: contains datablock(s) global, I. DOI: 10.1107/S1600536812045904/fy2074sup1.cif

Structure factors: contains datablock(s) I. DOI: 10.1107/S1600536812045904/fy2074Isup2.hkl

Supplementary material file. DOI: 10.1107/S1600536812045904/fy2074Isup3.cml

Additional supplementary materials:  crystallographic information; 3D view; checkCIF report

## Figures and Tables

**Fig. 1.**
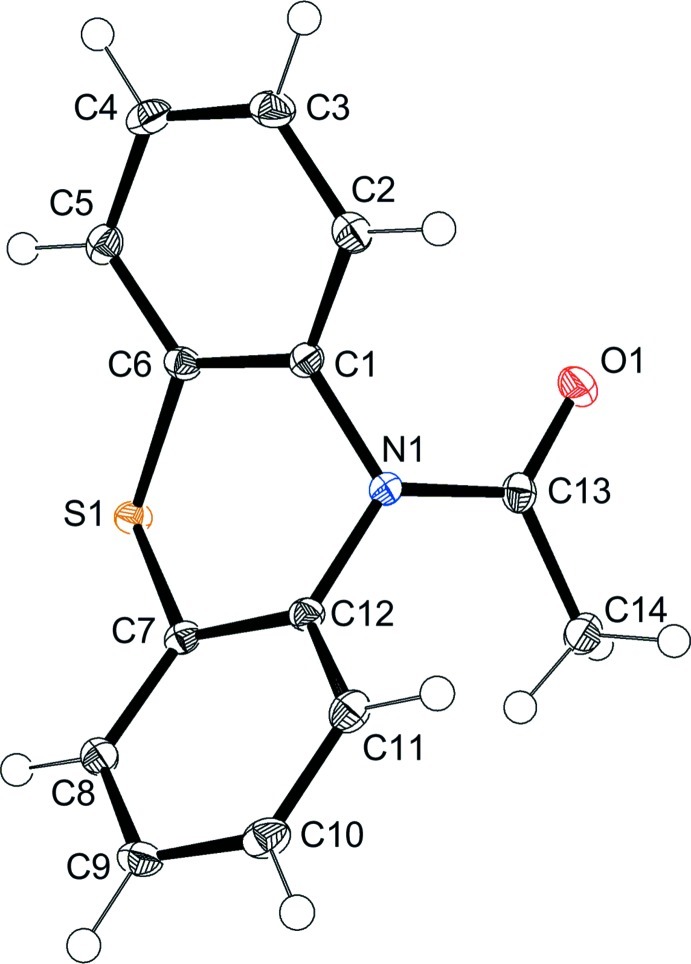
The asymmetric unit of the title compound with atom-numbering scheme. Displacement ellipsoids are drawn at the 50% probability level and H atoms are shown as small spheres.

## supplementary crystallographic information

### Comment

Phenothiazine derivatives have been interesting from the viewpoint of formation of charge-transfer complexes and biochemical reactivities. The title compound is an *N*-acetylated phenothiazine in which the orientation of the acetyl group is thought to determine the molecular structure and furthermore to affect the oxidation reactivity at 5-position.

The phenothiazine moiety has a butterfly structure, and the central six-membered ring has a boat form. The dihedral angle between the C1—C6 and C7—C12 planes is 133.61 (7)°, which is smaller than a usual angle (Chu & Van der Helm, 1974, 1975). The five atoms of N1, C1, C12, C13 and O1 lie on almost the same plane (the N1/C1/C12/Cl3/O1 plane: r.m.s. deviation = 0.0317 Å), showing efficient conjugation between the lone pair of N1 and the carbonyl group. The proximity of C11 and C14 is indicated by the intramolecular contact distance of 3.130 (3) Å, although little effective contact is observed around O1. The relatively small dihedral angle between the benzene rings is thought to reduce the steric repulsion between the phenothiazine moiety and the acetyl group. There are three reports concerning 1-(10*H*-phenothiazin-10-yl)ethanone derivatives (Meester & Chu, 1986; Wang *et al.*, 2009; Siddegowda *et al.*, 2011). Similar steric effects can also be recognized in these cases.

### Experimental

Single crystals with sufficient quality for X-ray crystallographical analysis were prepared by recrystallization from an ethanol solution.

### Refinement

The C-bound H atoms were placed at ideal positions and were refined as riding on their parent C atoms. *U*_iso_(H) values of the H atoms were set at 1.2*U*_eq_(parent atom).

### Crystal data

**Table:**

C_14_H_11_NOS	*F*(000) = 1008.00
*M**_r_* = 241.31	*D*_x_ = 1.421 Mg m^−^^3^
Monoclinic, *C*2/*c*	Mo *K*α radiation, λ = 0.71075 Å
Hall symbol: -C 2yc	Cell parameters from 3415 reflections
*a* = 21.435 (6) Å	θ = 2.1–31.1°
*b* = 8.897 (3) Å	µ = 0.27 mm^−^^1^
*c* = 12.738 (4) Å	*T* = 93 K
β = 111.753 (3)°	Block, colorless
*V* = 2256.2 (11) Å^3^	0.17 × 0.10 × 0.10 mm
*Z* = 8	

### Data collection

**Table:**

Rigaku Saturn724+ diffractometer	2337 reflections with *F*^2^ > 2σ(*F*^2^)
Detector resolution: 7.111 pixels mm^-1^	*R*_int_ = 0.025
ω scans	θ_max_ = 27.5°
Absorption correction: numerical (*NUMABS*; Rigaku, 1999)	*h* = −27→27
*T*_min_ = 0.969, *T*_max_ = 0.974	*k* = −11→8
9030 measured reflections	*l* = −16→16
2584 independent reflections	

### Refinement

**Table:**

Refinement on *F*^2^	Secondary atom site location: difference Fourier map
*R*[*F*^2^ > 2σ(*F*^2^)] = 0.031	Hydrogen site location: inferred from neighbouring sites
*wR*(*F*^2^) = 0.081	H-atom parameters constrained
*S* = 1.03	*w* = 1/[σ^2^(*F*_o_^2^) + (0.042*P*)^2^ + 2.3718*P*] where *P* = (*F*_o_^2^ + 2*F*_c_^2^)/3
2584 reflections	(Δ/σ)_max_ < 0.001
154 parameters	Δρ_max_ = 0.31 e Å^−^^3^
0 restraints	Δρ_min_ = −0.24 e Å^−^^3^
Primary atom site location: structure-invariant direct methods	

### Special details

**Table:**

Refinement. Refinement was performed using all reflections. The weighted *R*-factor (*wR*) and goodness of fit (*S*) are based on *F*^2^. *R*-factor (gt) are based on *F*. The threshold expression of *F*^2^ > 2.0 σ(*F*^2^) is used only for calculating *R*-factor (gt).

### Fractional atomic coordinates and isotropic or equivalent isotropic displacement parameters (Å^2^)

**Table:**

	*x*	*y*	*z*	*U*_iso_*/*U*_eq_	
S1	0.414831 (16)	0.27277 (3)	0.08674 (2)	0.01289 (10)	
O1	0.39200 (5)	−0.21234 (10)	0.18741 (8)	0.0181 (2)	
N1	0.37281 (5)	0.03491 (12)	0.20689 (9)	0.0120 (3)	
C1	0.32864 (6)	0.04797 (14)	0.09052 (10)	0.0118 (3)	
C2	0.27278 (7)	−0.04399 (14)	0.04325 (11)	0.0149 (3)	
C3	0.23329 (7)	−0.02967 (15)	−0.07063 (11)	0.0169 (3)	
C4	0.24838 (7)	0.07854 (15)	−0.13668 (11)	0.0163 (3)	
C5	0.30261 (7)	0.17472 (15)	−0.08875 (11)	0.0147 (3)	
C6	0.34325 (6)	0.15792 (14)	0.02484 (10)	0.0121 (3)	
C7	0.41033 (6)	0.29392 (14)	0.22130 (10)	0.0113 (3)	
C8	0.42569 (6)	0.43164 (14)	0.27746 (11)	0.0134 (3)	
C9	0.42106 (6)	0.44609 (15)	0.38294 (11)	0.0156 (3)	
C10	0.39899 (7)	0.32640 (15)	0.43056 (11)	0.0165 (3)	
C11	0.38202 (7)	0.19044 (15)	0.37316 (11)	0.0147 (3)	
C12	0.38932 (6)	0.17307 (14)	0.26952 (10)	0.0116 (3)	
C13	0.40672 (6)	−0.09848 (14)	0.24523 (11)	0.0132 (3)	
C14	0.46316 (7)	−0.09615 (15)	0.35876 (11)	0.0164 (3)	
H2	0.2617	−0.1161	0.0885	0.0179*	
H3	0.1957	−0.0941	−0.1038	0.0202*	
H4	0.2215	0.0866	−0.2148	0.0195*	
H5	0.3119	0.2512	−0.1330	0.0177*	
H8	0.4392	0.5146	0.2439	0.0161*	
H9	0.4330	0.5384	0.4229	0.0187*	
H10	0.3955	0.3376	0.5024	0.0198*	
H11	0.3655	0.1098	0.4044	0.0176*	
H14A	0.4889	−0.0030	0.3668	0.0197*	
H14B	0.4927	−0.1826	0.3651	0.0197*	
H14C	0.4447	−0.1013	0.4185	0.0197*	

### Atomic displacement parameters (Å^2^)

**Table:**

	*U*^11^	*U*^22^	*U*^33^	*U*^12^	*U*^13^	*U*^23^
S1	0.01464 (17)	0.01445 (17)	0.01035 (15)	−0.00323 (11)	0.00552 (12)	−0.00079 (11)
O1	0.0196 (5)	0.0115 (5)	0.0205 (5)	−0.0003 (4)	0.0043 (4)	−0.0018 (4)
N1	0.0141 (6)	0.0110 (5)	0.0098 (5)	−0.0003 (4)	0.0031 (5)	0.0002 (4)
C1	0.0123 (6)	0.0116 (6)	0.0110 (6)	0.0020 (5)	0.0038 (5)	−0.0008 (5)
C2	0.0141 (6)	0.0130 (6)	0.0176 (7)	−0.0003 (5)	0.0060 (5)	−0.0007 (5)
C3	0.0119 (7)	0.0161 (6)	0.0197 (7)	0.0003 (5)	0.0025 (6)	−0.0040 (5)
C4	0.0129 (6)	0.0198 (7)	0.0129 (6)	0.0048 (5)	0.0010 (5)	−0.0031 (5)
C5	0.0164 (7)	0.0147 (6)	0.0133 (6)	0.0035 (5)	0.0059 (5)	0.0009 (5)
C6	0.0113 (6)	0.0116 (6)	0.0132 (6)	0.0005 (5)	0.0045 (5)	−0.0022 (5)
C7	0.0098 (6)	0.0136 (6)	0.0101 (6)	0.0019 (5)	0.0034 (5)	−0.0005 (5)
C8	0.0108 (6)	0.0128 (6)	0.0157 (6)	0.0001 (5)	0.0039 (5)	−0.0003 (5)
C9	0.0135 (7)	0.0155 (6)	0.0156 (6)	0.0013 (5)	0.0027 (5)	−0.0052 (5)
C10	0.0163 (7)	0.0209 (7)	0.0122 (6)	0.0033 (6)	0.0052 (5)	−0.0019 (5)
C11	0.0143 (6)	0.0174 (7)	0.0131 (6)	0.0007 (5)	0.0058 (5)	0.0021 (5)
C12	0.0103 (6)	0.0116 (6)	0.0116 (6)	0.0008 (5)	0.0026 (5)	−0.0008 (5)
C13	0.0138 (6)	0.0128 (6)	0.0146 (6)	−0.0008 (5)	0.0070 (5)	0.0022 (5)
C14	0.0171 (7)	0.0163 (6)	0.0150 (7)	0.0014 (5)	0.0050 (6)	0.0020 (5)

### Geometric parameters (Å, º)

**Table:**

S1—C6	1.7673 (13)	C9—C10	1.392 (2)
S1—C7	1.7621 (15)	C10—C11	1.3903 (19)
O1—C13	1.2231 (16)	C11—C12	1.394 (3)
N1—C1	1.4376 (15)	C13—C14	1.5028 (17)
N1—C12	1.4365 (17)	C2—H2	0.950
N1—C13	1.3828 (16)	C3—H3	0.950
C1—C2	1.3897 (18)	C4—H4	0.950
C1—C6	1.396 (2)	C5—H5	0.950
C2—C3	1.3881 (18)	C8—H8	0.950
C3—C4	1.393 (3)	C9—H9	0.950
C4—C5	1.3893 (19)	C10—H10	0.950
C5—C6	1.3930 (17)	C11—H11	0.950
C7—C8	1.3953 (18)	C14—H14A	0.980
C7—C12	1.393 (2)	C14—H14B	0.980
C8—C9	1.389 (3)	C14—H14C	0.980
			
S1···N1	2.9418 (14)	C11···H14B^xiii^	3.4202
O1···C1	2.7367 (16)	C12···H4^v^	3.0777
O1···C2	2.9441 (16)	C13···H3^ii^	3.5525
O1···C12	3.5917 (18)	C13···H5^vii^	3.2746
C1···C4	2.7782 (18)	C13···H8^iii^	3.5131
C1···C7	2.9099 (17)	C13···H14A^i^	3.1820
C1···C11	3.581 (2)	C13···H14B^i^	3.0711
C2···C5	2.796 (3)	C14···H5^vii^	3.5612
C2···C12	3.5972 (18)	C14···H9^iii^	3.4709
C2···C13	3.1019 (17)	C14···H10^xiii^	3.5985
C3···C6	2.7741 (19)	C14···H14A^i^	3.4899
C6···C12	2.903 (2)	C14···H14A^xiii^	3.3758
C6···C13	3.4792 (19)	C14···H14B^i^	3.4086
C7···C10	2.780 (3)	C14···H14C^xiii^	3.2995
C7···C13	3.508 (2)	H2···C2^ii^	3.4044
C8···C11	2.795 (2)	H2···C3^ii^	3.1645
C9···C12	2.7789 (19)	H2···C10^ix^	3.4004
C11···C13	3.191 (2)	H2···H2^ii^	3.1902
C11···C14	3.130 (3)	H2···H3^ii^	2.7183
C12···C14	2.8638 (19)	H2···H4^vii^	2.9472
S1···C8^i^	3.5062 (15)	H2···H5^vii^	3.5148
O1···C3^ii^	3.4254 (18)	H2···H10^ix^	3.1583
O1···C8^iii^	3.3551 (17)	H3···O1^ii^	2.4806
O1···C10^iv^	3.481 (2)	H3···C2^ii^	3.3218
O1···C14^i^	3.522 (3)	H3···C7^v^	3.4668
C3···O1^ii^	3.4254 (18)	H3···C8^v^	3.0805
C3···C8^v^	3.5581 (18)	H3···C10^ix^	3.5790
C4···C7^v^	3.362 (2)	H3···C13^ii^	3.5525
C4···C8^v^	3.491 (2)	H3···H2^ii^	2.7183
C4···C12^v^	3.5717 (19)	H3···H5^xiv^	3.5725
C7···C4^v^	3.362 (2)	H3···H8^v^	2.8676
C8···S1^i^	3.5062 (15)	H3···H10^ix^	2.7814
C8···O1^vi^	3.3551 (17)	H4···C7^v^	2.9911
C8···C3^v^	3.5581 (18)	H4···C8^v^	2.9579
C8···C4^v^	3.491 (2)	H4···C9^v^	3.0259
C8···C8^i^	3.506 (3)	H4···C10^v^	3.0919
C10···O1^vii^	3.481 (2)	H4···C11^v^	3.0998
C12···C4^v^	3.5717 (19)	H4···C12^v^	3.0777
C13···C14^i^	3.503 (3)	H4···H2^iv^	2.9472
C14···O1^i^	3.522 (3)	H4···H5^xiv^	3.4855
C14···C13^i^	3.503 (3)	H4···H8^v^	3.4440
S1···H5	2.8571	H4···H9^v^	3.5674
S1···H8	2.8527	H4···H11^iv^	3.3789
O1···H2	2.7411	H5···O1^iv^	3.3489
O1···H14A	3.0798	H5···C2^v^	3.5950
O1···H14B	2.4958	H5···C9^viii^	3.5246
O1···H14C	2.9067	H5···C13^iv^	3.2746
N1···H2	2.6627	H5···C14^iv^	3.5612
N1···H11	2.6624	H5···H2^iv^	3.5148
N1···H14A	2.5881	H5···H3^xv^	3.5725
N1···H14B	3.2510	H5···H4^xv^	3.4855
N1···H14C	2.8334	H5···H9^viii^	3.0641
C1···H3	3.2575	H5···H11^iv^	3.3848
C1···H5	3.2778	H5···H14C^iv^	2.9882
C2···H4	3.2687	H8···O1^vi^	2.6273
C3···H5	3.2715	H8···C3^v^	3.5369
C4···H2	3.2716	H8···C8^i^	3.0975
C5···H3	3.2680	H8···C13^vi^	3.5131
C6···H2	3.2727	H8···H3^v^	2.8676
C6···H4	3.2601	H8···H4^v^	3.4440
C7···H9	3.2612	H8···H8^i^	2.5542
C7···H11	3.2720	H8···H10^viii^	3.1524
C7···H14A	3.3070	H8···H14B^vi^	3.1051
C8···H10	3.2705	H8···H14B^xvi^	3.5810
C9···H11	3.2690	H9···S1^x^	2.8168
C10···H8	3.2734	H9···O1^vi^	3.5684
C11···H9	3.2663	H9···C9^xi^	3.1910
C11···H14A	2.8911	H9···C10^xi^	3.5933
C11···H14C	2.8813	H9···C14^vi^	3.4709
C12···H8	3.2774	H9···H4^v^	3.5674
C12···H10	3.2657	H9···H5^x^	3.0641
C12···H14A	2.5664	H9···H9^xi^	2.8955
C12···H14C	3.0495	H9···H14B^vi^	3.0059
C13···H2	3.0122	H9···H14C^vi^	3.2172
C13···H11	3.1065	H10···O1^vii^	2.6337
C14···H11	2.9960	H10···C2^xii^	3.5929
H2···H3	2.3388	H10···C3^xii^	3.3992
H3···H4	2.3384	H10···C14^xiii^	3.5985
H4···H5	2.3425	H10···H2^xii^	3.1583
H8···H9	2.3410	H10···H3^xii^	2.7814
H9···H10	2.3377	H10···H8^x^	3.1524
H10···H11	2.3422	H10···H14B^xiii^	2.7382
H11···H14A	3.0286	H11···O1^vii^	3.5544
H11···H14C	2.4928	H11···C1^vii^	3.0992
S1···H9^viii^	2.8168	H11···C2^vii^	3.1669
S1···H14A^i^	3.1180	H11···C3^vii^	3.0516
S1···H14C^iv^	2.8892	H11···C4^vii^	2.9005
O1···H3^ii^	2.4806	H11···C5^vii^	2.8846
O1···H5^vii^	3.3489	H11···C6^vii^	2.9661
O1···H8^iii^	2.6273	H11···H4^vii^	3.3789
O1···H9^iii^	3.5684	H11···H5^vii^	3.3848
O1···H10^iv^	2.6337	H11···H14A^xiii^	3.5203
O1···H11^iv^	3.5544	H11···H14B^xiii^	3.4153
O1···H14A^i^	3.4300	H14A···S1^i^	3.1180
O1···H14B^i^	2.8025	H14A···O1^i^	3.4300
N1···H14A^i^	3.4407	H14A···N1^i^	3.4407
C1···H11^iv^	3.0992	H14A···C13^i^	3.1820
C2···H2^ii^	3.4044	H14A···C14^i^	3.4899
C2···H3^ii^	3.3218	H14A···C14^xiii^	3.3758
C2···H5^v^	3.5950	H14A···H11^xiii^	3.5203
C2···H10^ix^	3.5929	H14A···H14A^i^	3.1810
C2···H11^iv^	3.1669	H14A···H14A^xiii^	3.2490
C3···H2^ii^	3.1645	H14A···H14B^i^	3.5061
C3···H8^v^	3.5369	H14A···H14C^xiii^	2.7361
C3···H10^ix^	3.3992	H14B···O1^i^	2.8025
C3···H11^iv^	3.0516	H14B···C10^xiii^	3.0537
C4···H11^iv^	2.9005	H14B···C11^xiii^	3.4202
C5···H11^iv^	2.8846	H14B···C13^i^	3.0711
C5···H14C^iv^	3.0826	H14B···C14^i^	3.4086
C6···H11^iv^	2.9661	H14B···H8^iii^	3.1051
C6···H14C^iv^	3.0002	H14B···H8^xvii^	3.5810
C7···H3^v^	3.4668	H14B···H9^iii^	3.0059
C7···H4^v^	2.9911	H14B···H10^xiii^	2.7382
C8···H3^v^	3.0805	H14B···H11^xiii^	3.4153
C8···H4^v^	2.9579	H14B···H14A^i^	3.5061
C8···H8^i^	3.0975	H14B···H14B^i^	3.0623
C9···H4^v^	3.0259	H14C···S1^vii^	2.8892
C9···H5^x^	3.5246	H14C···C5^vii^	3.0826
C9···H9^xi^	3.1910	H14C···C6^vii^	3.0002
C10···H2^xii^	3.4004	H14C···C14^xiii^	3.2995
C10···H3^xii^	3.5790	H14C···H5^vii^	2.9882
C10···H4^v^	3.0919	H14C···H9^iii^	3.2172
C10···H9^xi^	3.5933	H14C···H14A^xiii^	2.7361
C10···H14B^xiii^	3.0537	H14C···H14C^xiii^	3.0883
C11···H4^v^	3.0998		
			
C6—S1—C7	98.29 (7)	O1—C13—C14	121.79 (11)
C1—N1—C12	115.85 (10)	N1—C13—C14	117.28 (11)
C1—N1—C13	119.51 (11)	C1—C2—H2	120.254
C12—N1—C13	123.31 (10)	C3—C2—H2	120.238
N1—C1—C2	122.11 (12)	C2—C3—H3	119.796
N1—C1—C6	117.79 (11)	C4—C3—H3	119.785
C2—C1—C6	120.10 (11)	C3—C4—H4	119.869
C1—C2—C3	119.51 (14)	C5—C4—H4	119.868
C2—C3—C4	120.42 (13)	C4—C5—H5	120.334
C3—C4—C5	120.26 (12)	C6—C5—H5	120.348
C4—C5—C6	119.32 (14)	C7—C8—H8	120.357
S1—C6—C1	119.23 (9)	C9—C8—H8	120.357
S1—C6—C5	120.44 (11)	C8—C9—H9	119.733
C1—C6—C5	120.32 (12)	C10—C9—H9	119.730
S1—C7—C8	120.32 (11)	C9—C10—H10	119.956
S1—C7—C12	119.31 (10)	C11—C10—H10	119.958
C8—C7—C12	120.34 (13)	C10—C11—H11	120.160
C7—C8—C9	119.29 (13)	C12—C11—H11	120.169
C8—C9—C10	120.54 (13)	C13—C14—H14A	109.468
C9—C10—C11	120.09 (15)	C13—C14—H14B	109.466
C10—C11—C12	119.67 (14)	C13—C14—H14C	109.474
N1—C12—C7	117.97 (13)	H14A—C14—H14B	109.477
N1—C12—C11	121.98 (13)	H14A—C14—H14C	109.471
C7—C12—C11	119.99 (12)	H14B—C14—H14C	109.472
O1—C13—N1	120.91 (11)		
			
C6—S1—C7—C8	−141.08 (9)	C2—C1—C6—S1	−179.67 (11)
C6—S1—C7—C12	36.99 (9)	C2—C1—C6—C5	−1.0 (2)
C7—S1—C6—C1	−37.65 (11)	C6—C1—C2—C3	2.6 (2)
C7—S1—C6—C5	143.66 (10)	C1—C2—C3—C4	−1.6 (3)
C1—N1—C12—C7	−48.72 (16)	C2—C3—C4—C5	−0.9 (3)
C1—N1—C12—C11	128.40 (12)	C3—C4—C5—C6	2.5 (3)
C12—N1—C1—C2	−131.80 (13)	C4—C5—C6—S1	177.10 (12)
C12—N1—C1—C6	47.89 (17)	C4—C5—C6—C1	−1.6 (2)
C1—N1—C13—O1	−11.7 (2)	S1—C7—C8—C9	179.29 (7)
C1—N1—C13—C14	166.77 (11)	S1—C7—C12—N1	0.64 (15)
C13—N1—C1—C2	60.91 (18)	S1—C7—C12—C11	−176.54 (7)
C13—N1—C1—C6	−119.41 (13)	C8—C7—C12—N1	178.71 (10)
C12—N1—C13—O1	−177.96 (13)	C8—C7—C12—C11	1.52 (17)
C12—N1—C13—C14	0.5 (2)	C12—C7—C8—C9	1.24 (17)
C13—N1—C12—C7	118.04 (14)	C7—C8—C9—C10	−2.34 (17)
C13—N1—C12—C11	−64.84 (18)	C8—C9—C10—C11	0.68 (18)
N1—C1—C2—C3	−177.76 (11)	C9—C10—C11—C12	2.10 (19)
N1—C1—C6—S1	0.64 (18)	C10—C11—C12—N1	179.74 (11)
N1—C1—C6—C5	179.34 (11)	C10—C11—C12—C7	−3.19 (18)

Symmetry codes: (i) −*x*+1, *y*, −*z*+1/2; (ii) −*x*+1/2, −*y*−1/2, −*z*; (iii) *x*, *y*−1, *z*; (iv) *x*, −*y*, *z*−1/2; (v) −*x*+1/2, −*y*+1/2, −*z*; (vi) *x*, *y*+1, *z*; (vii) *x*, −*y*, *z*+1/2; (viii) *x*, −*y*+1, *z*−1/2; (ix) −*x*+1/2, *y*−1/2, −*z*+1/2; (x) *x*, −*y*+1, *z*+1/2; (xi) −*x*+1, −*y*+1, −*z*+1; (xii) −*x*+1/2, *y*+1/2, −*z*+1/2; (xiii) −*x*+1, −*y*, −*z*+1; (xiv) −*x*+1/2, *y*−1/2, −*z*−1/2; (xv) −*x*+1/2, *y*+1/2, −*z*−1/2; (xvi) −*x*+1, *y*+1, −*z*+1/2; (xvii) −*x*+1, *y*−1, −*z*+1/2.

## References

[bb1] Chu, S. S. C. & Van der Helm, D. (1974). *Acta Cryst.* B**30**, 2489–2490.

[bb2] Chu, S. S. C. & Van der Helm, D. (1975). *Acta Cryst.* B**31**, 1179–1183.

[bb3] Farrugia, L. J. (2012). *J. Appl. Cryst.* **45**, 849–854.

[bb4] Meester, P. & Chu, S. S. C. (1986). *J. Heterocycl. Chem.* **23**, 1249–1252.

[bb5] Rigaku (1999). *NUMABS* Rigaku Corporation, Tokyo, Japan.

[bb6] Rigaku (2008). *CrystalClear* Rigaku Corporation, Tokyo, Japan.

[bb7] Rigaku (2010). *CrystalStructure* Rigaku Corporation, Tokyo, Japan.

[bb8] Sheldrick, G. M. (2008). *Acta Cryst.* A**64**, 112–122. 10.1107/S0108767307043930 18156677

[bb9] Siddegowda, M. S., Jasinski, J. P., Golen, J. A. & Yathirajan, H. S. (2011). *Acta Cryst.* E**67**, o1702. 10.1107/S1600536811021854 PMC3151987 21837098

[bb10] Wang, Q., Yang, L., Xu, Z. & Sun, Y. (2009). *Acta Cryst.* E**65**, o1978. 10.1107/S1600536809028487 PMC2977198 21583654

